# Wireframe DNA Origami for the Cellular Delivery of Platinum(II)-Based Drugs

**DOI:** 10.3390/ijms242316715

**Published:** 2023-11-24

**Authors:** Erik De Luca, Yang Wang, Igor Baars, Federica De Castro, Marco Lolaico, Danilo Migoni, Cosimo Ducani, Michele Benedetti, Björn Högberg, Francesco Paolo Fanizzi

**Affiliations:** 1 Dipartimento di Scienze e Tecnologie Biologiche ed Ambientali, Università del Salento, Prov.le Lecce-Monteroni, Centro Ecotekne, I-73100 Lecce, Italy; erik.deluca@unisalento.it (E.D.L.); federica.decastro@unisalento.it (F.D.C.); danilo.migoni@unisalento.it (D.M.); michele.benedetti@unisalento.it (M.B.); 2 Department of Medical Biochemistry and Biophysics, Karolinska Institutet, SE-17177 Stockholm, Sweden; yang.wang@ki.se (Y.W.); igor.baars@ki.se (I.B.); marco.lolaico@ki.se (M.L.); cosimo.ducani@ki.se (C.D.); bjorn.hogberg@ki.se (B.H.)

**Keywords:** DNA origami, drug delivery, platinum compounds, cisplatin, antitumor drug

## Abstract

The DNA origami method has revolutionized the field of DNA nanotechnology since its introduction. These nanostructures, with their customizable shape and size, addressability, nontoxicity, and capacity to carry bioactive molecules, are promising vehicles for therapeutic delivery. Different approaches have been developed for manipulating and folding DNA origami, resulting in compact lattice-based and wireframe designs. Platinum-based complexes, such as cisplatin and phenanthriplatin, have gained attention for their potential in cancer and antiviral treatments. Phenanthriplatin, in particular, has shown significant antitumor properties by binding to DNA at a single site and inhibiting transcription. The present work aims to study wireframe DNA origami nanostructures as possible carriers for platinum compounds in cancer therapy, employing both cisplatin and phenanthriplatin as model compounds. This research explores the assembly, platinum loading capacity, stability, and modulation of cytotoxicity in cancer cell lines. The findings indicate that nanomolar quantities of the ball-like origami nanostructure, obtained in the presence of phenanthriplatin and therefore loaded with that specific drug, reduced cell viability in MCF-7 (cisplatin-resistant breast adenocarcinoma cell line) to 33%, while being ineffective on the other tested cancer cell lines. The overall results provide valuable insights into using wireframe DNA origami as a highly stable possible carrier of Pt species for very long time-release purposes.

## 1. Introduction

The idea of using nucleic acids as building blocks for the construction of functional structures and materials was originally conceived by Nadrian Seeman in 1982 [1]. Notwithstanding, only after its introduction in 2006 by Paul Rothemund, the DNA origami method drastically expanded the DNA nanotechnology field [2]. DNA origami is based on using hundreds of short oligonucleotides (called staples) to hybridize to different regions of a long single-stranded DNA scaffold and thus fold the designed structure during a thermal ramp. During the folding of DNA origami, the specificity of Watson–Crick base pairing is the mechanism to control matter at the nanoscale [1,3]. Different methods for manipulating and designing DNA origami emerged, leading to obtaining complex circular structures, spherical balls, or other shapes [3,4].

DNA origami structures provide a versatile engineering platform that ensures the homogenous size, shape, and charge for each particle [5]. Their addressability endows them with the unique advantage of presenting biofunctional components with nanometric precision [6]. Furthermore, DNA origami’s low toxicity has allowed their use for the targeted transport of small molecular drugs, proteins, aptamers [7], nanoparticles, antibodies [8], or quantum dots [9] to cells. Specifically, doxorubicin, which can intercalate into grooves of DNA [10], has been widely used for drug delivery experiments with DNA origami nanostructures [11,12]. When doxorubicin is loaded in a DNA origami structure, studies have reported increased apoptosis in various cancer cell lines [13]. Moreover, the cellular elimination rate of the free drug is faster than the doxorubicin retained inside the nanostructures, which, on the contrary, diffuses out gradually [5]. The use of a DNA origami carrier has also shown the capability to circumvent drug resistance in various cell lines [14,15].

Since the FDA approved cisplatin (*cis*-[PtCl₂(NH₃)₂]) in 1978, as an anticancer drug, there has been a significant increase in the interest of platinum-based complexes [16]. In addition to their antitumor properties, other applications, such as antiviral applications, have been discovered [17]. Thousands of new platinum compounds have been synthesized in an effort to reduce resistance and side effects associated with treatments [18]. Sala et al. recently used cisplatin as both a load and stabilizer for a two-dimensional DNA origami sheet [19]. Through the mechanism by which each cisplatin molecule can covalently bind to one or two adjacent purine bases (with a marked preference for guanines) in the DNA strands, the origami sheet could be cross-linked. On FaDu cells (hypopharyngeal carcinoma), it was found that the release of cisplatin and/or other active species from the origami sheet proceeded slowly [19]. The origami nanostructures used in this and other studies were the compact lattice-based type, which require tens of millimolar cations, such as magnesium or sodium, for correct folding and stability [20]. This creates a risk of potential disassembly of the origami structures when applied in biological systems [21].

In contrast, wireframe-type origami nanostructures have a lower DNA packaging density and, therefore, are stable in low-cationic buffers [22,23]. This characteristic leads to a higher local deformability of the structures, and it has been found to be involved in the interaction of DNA origami with surface moieties on cells. Wireframe rod-like DNA origamis are more likely to attach to the cell surface, rather than being internalized, while compact DNA origamis were internalized into cells to a larger extent. Wireframe DNA origami displays a higher penetration ability in cell spheroid tissue models than compact DNA origami, probably related to differences in uptake dynamics [21].

Both the DNA origami nanostructure types offer ease of synthesis and conjugation of functional moieties to target the release of cargo [24].

*Cis*-[Pt(NH_3_)_2_Cl(phenanthridine)]^+^, phenanthriplatin, a monofunctional platinum(II) complex, has shown significant antitumor potential, and it is seven to forty times more cytotoxic than cisplatin in different cancer cells [25,26]. Mechanistically, being distinct from the preferential two-site binding to the *N7* position of purines observed for cisplatin when bonded to DNA, phenanthriplatin binds DNA at a single site, specifically targeting the *N*7 position of guanine, leading to little distortion in the double helix and the inhibition of transcription [27]. Theoretically, this difference can affect, in addition to the stability of the metal containing nanostructures, their Pt loading and release profiles. The present work aims to study if three-dimensional lattice-based and wireframe DNA origami nanostructures could be suitable for the delivery of platinum compounds for therapeutic purposes. More specifically, cisplatin and phenanthriplatin were used as model compounds for the first investigations, focusing on DNA origami nanostructure stability and modulation of cytotoxicity in cancer cell lines. The use of DNA origami as potential carrier for the delivery of platinum-based drugs may hold the key to overcoming the intrinsic or acquired resistance to platinum drugs observed in some tumors [28]. Although previous work reported the simple loading of cisplatin on a folded two-dimensional DNA origami sheet [19], in the present work, the final nanostructures were folded using Pt drugs in the presence of origami precursors “scaffold” and with the help of specific oligo-DNA building blocks (staples). In this respect, this investigation could be also described as one of the first attempts aiming at the construction of a self-assembled DNA-based Pt drugs release system.

## 2. Results and Discussion

### 2.1. Origami Structures for the Loading of Platinum Compounds

**Figure 1:**
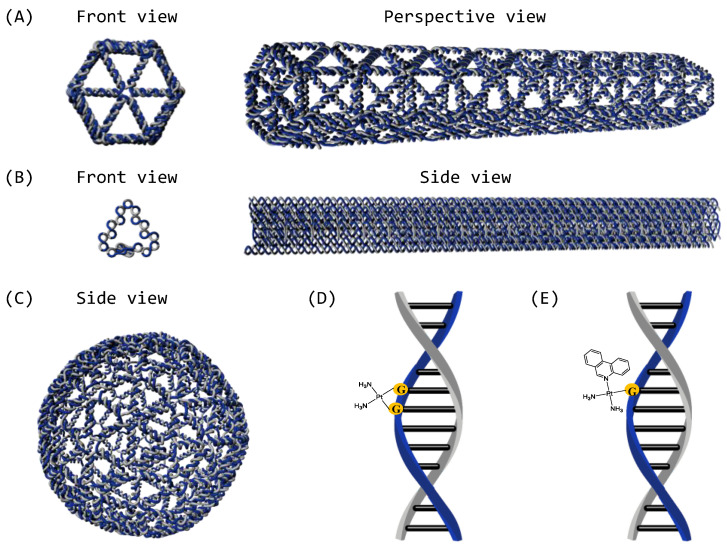
Rendering of the considered DNA origami nanostructures; visualization through Blender 3.5 software (scaffold is colored in blue, and staples are colored in gray). (**A**) Hexagonal rod. (**B**) 18HB. (**C**) The ball. Schematic representation of the main mono- and bis-adducts with DNA, considered responsible for antitumor activity of cisplatin (**D**) and phenanthriplatin (**E**) drugs.

Two three-dimensional wireframe DNA origami nanostructures, with their scaffold DNA routed along the edges of the predesigned triangulated polygons, were designed in previous work with vHelix [29]. These consist of the hexagonal rod (HR) [30], a hollow rod-like structure, which has a length of approximately 140 nm and a diameter of 23.2 nm (Figure 1A); the ball [29], which has 92 verts, 270 edges, 180 faces, an average length of 10.2 nm for each edge, and a diameter of 66.53 nm. (Figure 1C). Meanwhile, a close-packed-style DNA origami, the 18-helix bundle (18HB) [5], previously designed with caDNAno with parallel DNA helices packed in a honeycomb lattice, was also prepared. The 18HB has a length of approximately 140 nm and a cross-section of around 11 nm (Figure 1B).

Mechanistically, the way that platinum-based anticancer drugs work inside cells is via biding to DNA by forming inter- and intra-DNA strand cross-links which interfere with DNA replication and transcription, causing cell cycle arrest and cell apoptosis or necrosis [31]. We hypothesized that a similar binding mechanism to origami in vitro could be used to load DNA origami nanostructures. In this work, we focus on two compounds: cisplatin and phenanthriplatin. In theory, one cisplatin molecule can covalently bind to the *N7* position of one or two purine residues of DNA (Figure 1D), while one phenanthriplatin can only bind to the *N7* of a single guanine [32] (Figure 1E).

### 2.2. Characterizations of the Platinum Complexes-Loaded Origami

**Figure 2:**
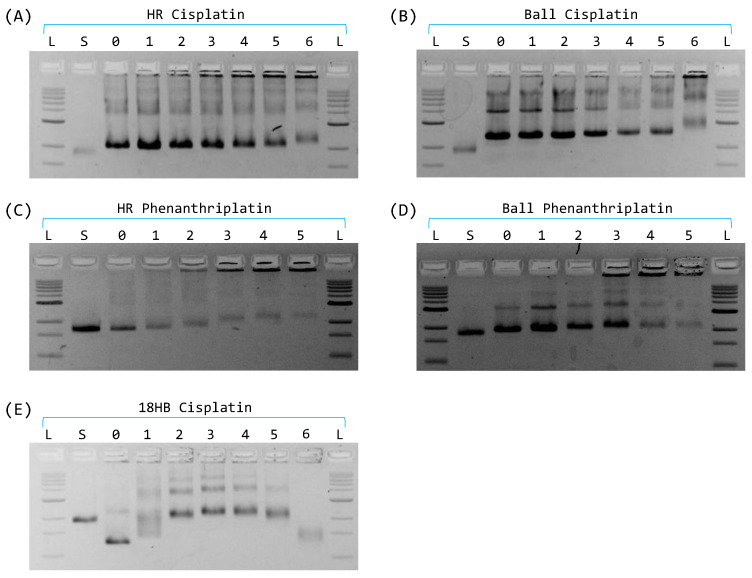
Agarose gel representing the folding of the considered structures with increasing amounts of cisplatin and phenanthriplatin, up to 250 µM. L = ladder; S = scaffold; 0 = DNA origami; 1 = structure folded with 16 µM; 2 = structure folded with 32 µM; 3 = structure folded with 64 µM; 4 = structure folded with 96 µM; 5 = structure folded with 128 µM; 6 = structure folded with 250 µM. (**A**) Hexagonal rod screening with cisplatin. (**B**) The ball screening with cisplatin. (**C**) Hexagonal rod screening with phenanthriplatin. (**D**) The ball screening with phenanthriplatin. (**E**) 18HB screening with cisplatin.

The DNA origami nanostructures were firstly characterized by electrophoretic mobility shift assay using 2% agarose gels. The empty DNA origami nanostructures (structures without loaded platinum complex) showed different migration speeds than the single-stranded scaffold DNA, verifying the successful preparations of the designed structures. Origami dimers, aggregates, and non-incorporated DNA strands (excessive staples) could also be resolved on the gels. The same electrophoretic assay was also used to monitor the incorporation of the platinum complexes. Compound loading was obtained by cisplatin or phenanthriplatin addition (in a concentration range between 16 µM and 250 µM) to the mixture of DNA materials before folding. A specific compound concentration-dependent trend of origami mobility shift was displayed on agarose gels (Figure 2), confirming the incorporation of both platinum compounds in the considered origami HR and ball. On the contrary, the latticed-based origami 18HB bands did not show a similar trend on the gels, hinting that platinum compounds could inhibit the correct assembly of the DNA strands into origami (Figure 2E).

**Figure 3:**
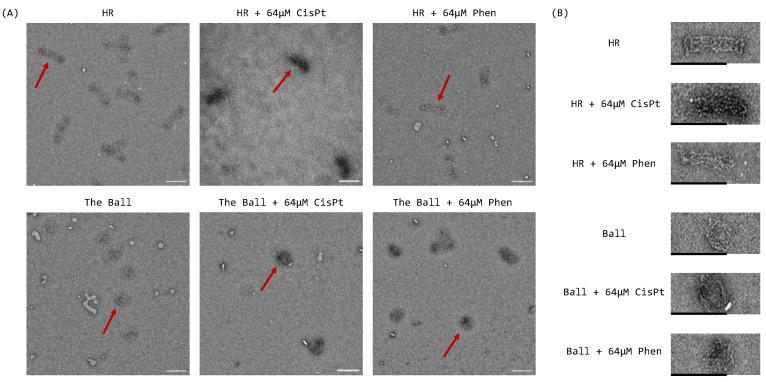
(**A**) Negative staining TEM of the considered DNA origami and comparison with the same structures folded with 64 µM of cisplatin and phenanthriplatin (red arrows show the selected DNA origami for the cropped images). (**B**) Cropped HR and ball structures. Scale bars are 100 nm.

The morphology of DNA origami nanostructures was characterized using negative-stain transmission electron microscopy (TEM). The sizes from TEM estimation were a bit smaller than the design sizes, because the origami samples were negative-stained and dried for the analysis. Compared with the empty origami, the HR and ball folded from platinum-containing (from 64 to 128 µM) buffers kept their rod- or ball-like shapes. Their folding generally did not seem to be obviously affected by the cisplatin loading, even at the highest concentrations (Figure 3 and Figure S1). In a molecular dynamics calculation analysis study, it was reported that phenanthriplatin induces prominent conformational distortions on the surrounding nucleotides that is higher than cisplatin [26]. Consistently, in our study, at high concentrations (96–128 µM) of phenanthriplatin, the correct folding efficiency of the wireframe origami decreased considerably, especially for the HR.

**Figure 4:**
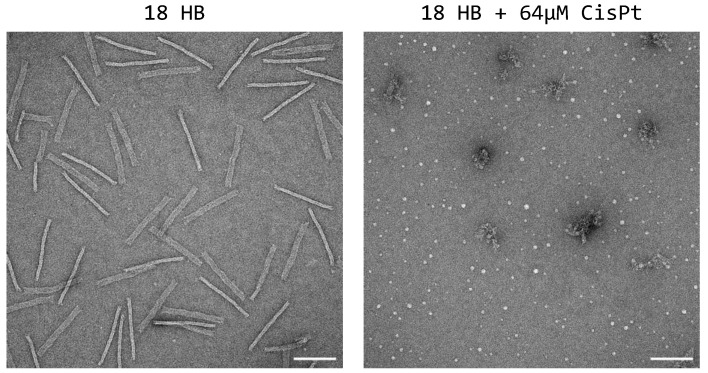
Negative staining TEM of the 18HB and comparison with the same structures folded with 64 µM of cisplatin. Scale bars are 100 nm.

The addition of the platinum complex was problematic for the folding of 18HB, inhibiting the correct assembly of the DNA materials into its rod-like shape (Figure 4). This indicated that the folding of this lattice type origami was more sensitive to the incorporation of platinum complexes than the wireframe type origami. A recent study has demonstrated that folded two-dimensional single-layer sheet lattice origami can be loaded with a platinum complex [19]. On the other hand, it should be noted that in the present case, differently from the reported procedure, the cisplatin was added during the folding. Moreover, the 18HB used in this work is three-dimensional, and the DNA is more densely packed than in a one-layer sheet of the previously reported successful result. In light of the inhibited proper folding of platinum-loaded 18HBs, this structure was not included in further experiments, and we only focused on the wireframe DNA origami nanostructures for the studies of possible incorporation and delivery of platinum complexes.

### 2.3. Loading Capacity of Platinum Complex in DNA Origami

**Figure 5:**
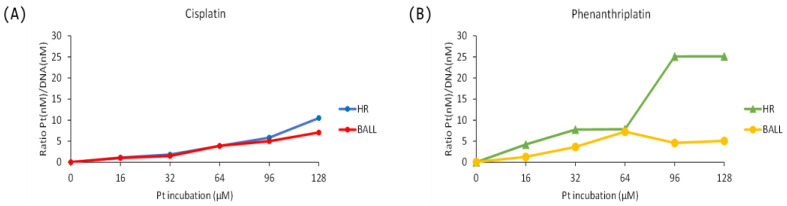
Loading capacity of HR and ball structures, stated as the ratio between the platinum content (nM) and the concentration of DNA (nM), incubated in the range of 0–128 µM of platinum complexes. (**A**) Cisplatin. (**B**) Phenanthriplatin.

The loading capacity of the DNA origami nanostructures was measured using inductively coupled plasma atomic emission spectroscopy (ICP-AES) analysis. During the origami folding, we always kept DNA materials at the same amount while changing the concentrations of platinum complexes in the folding buffer from 0 to 128 µM. For cisplatin, the loading profile showed a dose-dependent linear progression for both the HR and ball. In addition, there was no cisplatin loading difference between the HR and ball when the concentrations of the compound were 96 µM or less. When the cisplatin concentration was 128 µM, HR showed a higher loading capacity that the ball. Phenanthriplatin displayed a higher binding efficiency than cisplatin. In particular, the resulting amount in the HR could measure up to fivefold higher than in the ball (Pt incubation 96 µM, Figure 5B). However, as shown in the TEM images (Figure S1), high concentrations of phenanthriplatin caused a significant distortion, especially for the HR structure, as the structures were overloaded and lose their proper folded conformation.

Based on the TEM and ICP-AES analysis, we decided to use 64 µM of cisplatin or phenanthriplatin as the optimal concentration to prepare the drug-loaded HR and ball for the following cellular experiments. Even though the cisplatin-loaded nanostructures were stable at higher platinum concentration in respect to phenanthriplatin, we decided to use the same concentration in order to perform a comparative study between the two platinum complexes and their interaction with the folding/loading of the two wireframe structures. Notwithstanding the use of the same folding buffer, scaffold, staples, and platinum drug concentrations for the incubation processes (64 µM), a lower amount of platinum/DNA concentration ratio was observed for cisplatin loading (about three) with respect to phenanthriplatin (of about six) (Figure 5).

### 2.4. Stability and Degradation Assay

Compared to compact lattice-based DNA origami, wireframe origami nanostructures are generally more stable in physiological buffers like PBS or cell culture medium. In the former, ~5–20 mM divalent cations (for example, Mg^2+^) are also required for structural integrity maintenance in order to overcome electrostatic repulsion between closely packed DNA phosphate anions [33]. Nevertheless, stability in physiological condition was investigated by evaluating the survival time of platinum complex-loaded HR and ball in cell culture medium by incubation in DMEM containing 10% heat-inactivated fetal bovine serum (FBS). Gel electrophoresis results showed that, at least for 72 h, the origami bands of both the bare origami and cisplatin or phenanthriplatin-loaded origami barely changed. This reveals that the wireframe origami structures can reliably survive up to 3 days in the condition of cell culture (Figure S2). On the other hand, the DNA origami nanostructures are sensitive to DNase I digestion [34,35], which challenges their biomedical applications when exposed to biofluids. We were interested in studying the possible effect of platinum complexes’ covalent binding to wireframe DNA origami on changing the sensitivity of these nanostructures to DNase I. We therefore incubated the origami structures with 0.36 U/mL of DNase I, which is the concentration of DNase I in blood [36], for different time periods. The performed experiment showed, for both the bare origami and the platinum complex-loaded origami, similar sensitivity to DNase I, with complete degradation occurring within less than 4 h (Figure S3).

### 2.5. In Vitro Assays on Immortalized Cultured Cancer Cells

The cytotoxicity of the cisplatin- or phenanthriplatin-loaded HR and ball origami was evaluated on three different cancer cell lines: lung adenocarcinoma (A549 cells), cervical adenocarcinoma (HeLa cells), and cisplatin-resistant breast adenocarcinoma (MCF-7 cells) [37]. We treated these cells with free drugs (cisplatin, phenanthriplatin), bare origami, or drug-loaded origami. Cell viability was further determined by luminescent cell viability assay at 24, 48, and 72 h. The cytotoxicity evaluations related to the use of origami structures (both free and Pt-loaded) were limited by the maximum allowed concentration for the origamis in order to preserve their structures. It is known that high concentration can enhance aggregation due to stacking interactions or structural interlocking of certain types of 3D DNA origami. Aggregation is also enhanced by redispersion of DNA origami in low buffer volumes after centrifugation if high concentrations are required [38]. Therefore, in our cytotoxicity evaluations, ranges of origami concentrations not exceeding 236 and 177 nM for HR and ball nanostructures, respectively, were used. It should be also considered that the maximum Pt loading capacity for the origami structures introduced a further limitation related to the highest amount of drug potentially administrable to the cell cultures as loaded compound in comparison with the free drug. According to this further limitation, the ranges of drugs evaluated in cell toxicity studies did not exceed 3 μM and 6 μM for cisplatin and phenanthriplatin, respectively.

The empty HR and ball nanostructures themselves did not show cytotoxicity within the ranges of considered concentrations and incubation periods. Neither cisplatin nor Pt-loaded HR and ball origamis, in the range of concentration allowed for loaded HR and Ball nanostructures, displayed any cytotoxicity (Figure S4). This result is consistent with the data reported in the literature related to the use of the free drug (cisplatin) in the same low concentration range (0.1–3 μM) [25].

**Figure 6:**
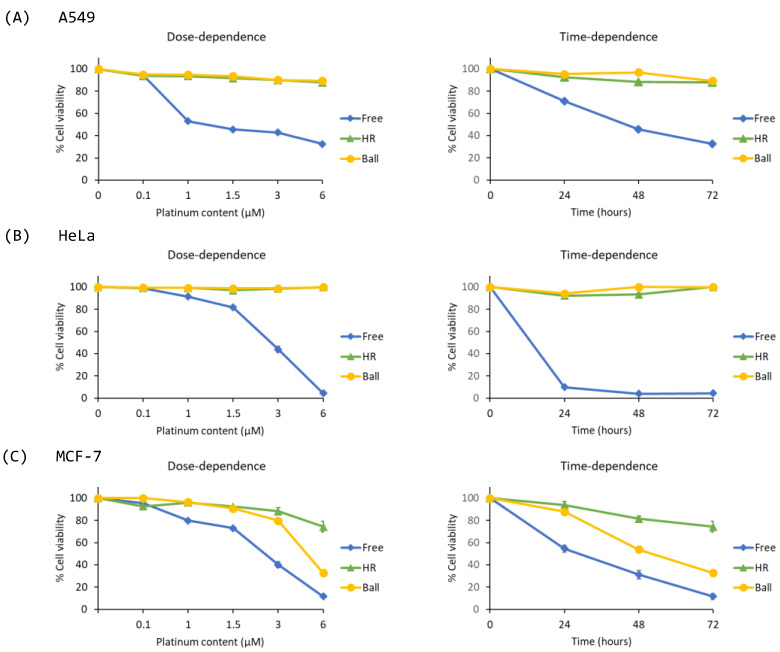
Left panel, dose-dependence at 72 h. Right panel, time-dependence at 6 µM of phenantriplatin-loaded nanostructures. (**A**) A549, lung adenocarcinoma cells. (**B**) HeLa, cervical adenocarcinoma cells. (**C**) MCF-7, breast adenocarcinoma cells.

Phenanthriplatin, used as free drug, was shown to be the most cytotoxic for all three different cancer cells lines, in accordance with data from the literature [39,40,41]. Cells were also treated with various concentrations (0.1–6 μM) of HR-phen or ball-phen (nanostructures loaded with phenanthriplatin) in comparison with free phenanthriplatin (Figure 6). For phenanthriplatin, we also performed a dose-dependent cytotoxicity assay within the above discussed limits for free drug and Pt-loaded concentrations, with an incubation time of 72 h. As expected, free phenanthriplatin showed a dose-dependent cytotoxic response for all the used cell lines at 72 h incubation times [39,40,41]. The cytotoxic activity was also confirmed in a time course experiment after 24 h incubation at the highest drug concentration (Figure 6). The most prominent cytotoxic effect was observed on the HeLa cancer cell lines, then on MCF-7 and A549. Interestingly, in sharp contrast with the free drug used at the same concentrations, loaded structures generally did not analogously decrease the cell viability in the dose dependency experiments nor did they show cytotoxicity at 24 h in the time course experiments. This supports the previously discussed structure stability results in the cell culture media. Only for the highest used concentration, at 72 h incubation time, the two phenanthriplatin-loaded nanostructures showed a detectable cytotoxic effect, selectively on the cisplatin-resistant MCF-7, among the three considered cell lines. Moreover, the ball-phen proved to be more effective than HR-phen (~32% of cell viability against the ~74%), with an IC_50_ at the 72 h of 4.692 ± 0.099 μM. This latter result may be related to a minimal amount of active drug derivatives released by possible origami degradation at the highest used origami concentration [38]. Remarkably, these active phenanthriplatin drug derivatives appear to be more effective on MCF-7 rather than other investigated cell lines. A specific sensitivity may characterize this cisplatin-resistant strain [26].

## 3. Materials and Methods

### 3.1. Structure Preparation

Scaffold DNA, including p7560 and p8064, was extracted and purified from M13 phage variants according to previous works [29]. Staple DNA oligos were purchased from Integrated DNA Technologies (Newark, NJ, USA). They were delivered desalted in water in 96-well plates at a concentration of 100 µM each. The staple strands were pooled and diluted with water to a working concentration of 400 nM each. Lists of staple strand sequences are found in previous work [21]. Hexagonal rod (HR) and ball were designed using vHelix [29]. Structures were folded in PBS (phosphate-buffered saline, pH 7.4) by an annealing program (80 °C for 5 min followed by cooling from 80 °C to 60 °C over 20 min and then a slow cooling from 60 °C to 24 °C over 14 h) with, respectively, p7560 and p8064, scaffold DNA (20 nM), and each staple DNA (100 nM). Removal of excess staples was performed by washing (repetitive concentration/dilution) the structures with their folding buffer in 100 kDa molecular weight cut-off 0.5 ml Amicon centrifugal filters (Merck KGaA, Darmstadt, Germany). In the second part of the work, in order to concentrate the samples, they were washed in Amicon^®^ Ultra-4 Centrifugal Filter Unit UFC810024 (Sigma-Aldrich Chemie GmbH, Steinheim, Germany).

### 3.2. Platinum Loading

Cisplatin and Phenanthriplatin were purchased from Sigma-Aldrich. The platinum complex (range between 16 and 250 μM) was added to the scaffold and staple DNA mixture before folding. After folding, structures were purified and concentrated by the ultrafiltration method. Briefly, the sample was transferred from the PCR tube to an Amicon 100 K filter tube (Millipore-Sigma, Burlington, MA, USA) and then diluted to 500 μL with the folding buffer. Then, it was centrifuged at 8000× *g* for 2 min, and the flowthrough was discarded. After repeating this process 6 times, the purified and concentrated structures were collected. As a higher concentration was needed for the in vitro cytotoxic assay, the samples were washed in Amicon^®^ Ultra-4 Centrifugal Filter Unit (Sigma-Aldrich, UFC810024) in order to be further concentrated.

### 3.3. Agarose Gel Electrophoresis

Agarose (Sigma-Aldrich) was used to prepare 2% agarose gels in 0.5× TRIS/borate/EDTA (TBE buffer) supplemented with 10 mM MgCl_2_ and 0.5 mg ml^−1^ ethidium bromide (Sigma-Aldrich). Within the ice water bath, gels were run in 0.5× TBE buffer supplemented with 10 mM MgCl_2_ at 90 volts for 3 h. After running, gels were imaged under the corresponding imaging channel using a GE LAS 4000 imager (GE Healthcare Europe GmbH, Freiburg, Germany).

### 3.4. Negative-Stain TEM

A 3 μL aliquot of 5 nM structure sample was spotted on a glow-discharged, carbon-coated, formvar resin grid (Electron Microscopy Sciences, Hatfield, PA, USA) for 20 s before blotting on a filter paper and then stained with aqueous uranyl formate solution (2% (*w*/*v*)). The stained sample was imaged using a Talos L120 C transmission electron microscope at 120 kV (Thermo Fisher Scientific, Waltham, MA, USA).

### 3.5. ICP-AES Measurements

To determine that amount of platinum, each sample was previously treated with 0.5 mL of 67% Suprapur^®^ nitric acid. The samples were then diluted with Suprapur^®^ water to a 5 mL final volume in order to obtain a suitable dilution of acid used for the mineralization process and to avoid damage to the system. Before injection into the instrument, each sample was filtered (0.45 μm) to eliminate the possible residues of the digestion process. The platinum concentration in analyzed samples was determined by a Thermo Fisher Scientific iCAP 6300 Duo ICP-AES spectrometer. The spectrophotometer was calibrated with a calibration line consisting of four points, each one corresponding to Pt concentration of 1 μg/L, 10 μg/L, 100 μg/L, and 1000 μg/L.

### 3.6. Degradation Assays by Gel Electrophoresis with Nucleases

DNase I (New England Biolabs, Ipswich, MA, USA) was diluted in the 1X PBS buffer and added at concentrations of 0.36 U ml^−1^, which is the average concentration in human blood [36]. The samples were incubated from 4 h to 72 h at 37 °C and then immediately loaded in a 2% agarose gel supplemented with 10 mM MgCl_2_ and run for 3 h at 90 V.

### 3.7. Cell Culture Media Stability Assay

The samples were diluted to possess 5 nM of structures for each condition. Then, they were added into DMEM (Dulbecco’s Modified Eagle’s Medium, Thermo Fisher Scientific S41965039) + 10% FBS. The samples were incubated at 37 °C for up to 72 h. After the incubation, the samples (10 μL) were taken for electrophoresis in agarose gel (2% (*w*/*v*) run for 3 hr at 90 V). GE LAS 4000 imager was used to image the gel.

### 3.8. Cell Culture

MCF7, HeLa, and A549 cells were obtained from ATCC (American Type Culture Collection, Manassas, VA, USA). The cell lines were cultured in DMEM containing 10% heat-inactivated FBS (Sigma F9665-500 ML) and 100 U/mL penicillin-streptomycin (Thermo Fisher Scientific 15140148) in a humidified environment of 5% CO_2_ at 37 °C.

### 3.9. Cell Viability Assay

For the cell viability assay, we used the CellTiter-Glo^®^ Luminescent Cell Viability Assay (Promega G7570, Madison, WI, USA). The cells were plated into 96-well white polystyrene plates (Thermo Fisher Scientific 136101), each well containing 100 µL DMEM medium and 40,000 cells. The plates were left in culture for 24 h. Following this, the medium was aspirated, and 90 µL of fresh medium was added. The total amount of platinum compounds and the DNA concentration were determined, respectively, by ICP-AES and nanodrop spectrophotometry. The cells were treated by adding, in each well, 10 µL of different concentrations of cisplatin-loaded DNA origami nanostructures and free cisplatin (in order to obtain a final range concentration from 0.1 to 3 µM), phenanthriplatin-loaded DNA origami structures and free phenanthriplatin (in order to obtain a final range concentration from 0.1 to 6 μM), and empty DNA origami structures solution (final concentration 30 nM). The wells treated with 10 µL of PBS represented the control. Following treatment, cells were incubated for 24, 48, and 72 h, followed by the CellTiter-Glo^®^ Luminescent Cell Viability Assay according to the provided protocol. Briefly, cells were removed from the incubator and left to equilibrate to room temperature for 30 min. After this, 100 µL of CellTiter-Glo reagent (Promega) was added to the wells, and the cells were mixed on an orbital shaker for 2 min. The plate was then incubated for 10 min at room temperature to stabilize the luminescence signal, and this signal was subsequently recorded on a multimode microplate reader (Varioskan LUX, Thermo Fisher Scientific). The viability was calculated compared to the PBS sample: % viable cells = (luminescence_sample_/luminescence_PBS_) × 100.

## 4. Conclusions

In this work, we demonstrated that platinum complexes can be successfully loaded on three-dimensional wireframe DNA origami during folding without significant impact on their structure. In contrast, the assembly of three-dimensional compact lattice-based rod origami could not be obtained using the tested platinum complexes. Furthermore, our data revealed that the binding of cisplatin or phenanthriplatin does not increase the stability of wireframe DNA origamis in DNase I-containing biofluids, while the Pt-loaded structures were quite stable for many hours in cell culture media without DNase I. The optimal conditions for the loading of the platinum drugs, without affecting the proper folding of the structures, were determined. Conformational changes were observed in TEM analysis after the addition of high concentrations of platinum complexes, especially for phenanthriplatin. Nevertheless, the overall 3D structure can still be maintained for both drugs in the presence of a Pt drug concentration not exceeding 64 μM. The binding of platinum drugs to the HR and ball was also confirmed through agarose gel electrophoresis and ICP-AES. The cytotoxicity of all tested platinum drug-loaded structures was evaluated on cancer cell lines in order to investigate their potential as carriers. In this respect, a limitation to the cytotoxicity tests was produced by the maximum allowed concentration for the origamis in order to preserve their structures. Consistently with the data in the literature, free cisplatin and the cisplatin-loaded DNA origami nanostructures did not show any cytotoxic effect in the extremely low and narrow range of allowed concentration for all considered cell lines [25]. On the other hand, free phenanthriplatin showed its well-established superior anticancer activity if compared to cisplatin, also at low concentrations [42]. In contrast with the free drug used at the same concentrations, phenanthriplatin-loaded structures generally did not decrease the cell viability analogously in the dose dependence experiments; neither showed cytotoxicity at 24 h in the time course experiments. The two HR and ball phenanthriplatin-loaded nanostructures only showed a detectable cytotoxic effect for the highest used concentration at 72 h incubation time and selectively on the cisplatin-resistant MCF-7 cell lines. The ball-phen with an IC_50_ at the 72 h of 4.692 ± 0.099 μM also proved to be more effective than HR-phen. The detectable cytotoxicity observed for ball-phen and HR-phen may be related to a minimal amount of active drug derivatives released by possible origami degradation at the highest used origami concentration [38]. These active phenanthriplatin drug derivatives appear to be selectively effective on MCF-7 rather than other considered cells, therefore requiring further characterization and specific investigation. Interestingly, rather than dealing with drug loading on completely folded nanostructures [19], this research focuses on the self-assembly of a DNA-based Pt drug release system starting from metal complexes and polynucleotides’ “scaffold” and “staples”, therefore representing a novelty in the field. The overall results of the present work suggest a possible use of these origami-loaded Pt species as highly stable possible carriers for very long time-release purposes. On the other hand, the possible drug derivatives released from these systems, such as those observed for phenanthriplatin and the selective cytotoxic activity on MCF-7 cell lines of ball-phen and HR-phen, despite the further work required, appear promising.

## Data Availability

Data are contained within the article or the Supplementary Materials.

## Supplementary Materials

The following supporting information can be downloaded at: https://www.mdpi.com/article/10.3390/ijms242316715/s1.
