# Supplementary material for: Wireframe DNA Origami for the Cellular Delivery of Platinum(II)-Based Drugs
Source: Int J Mol Sci. 2023 Nov 24;24(23):16715. doi: 10.3390/ijms242316715 (PMC10706596; doi:10.3390/ijms242316715)
Supplement: Supplementary file 1 [file ijms-24-16715-s001.zip › ijms-2727618-supplementary.pdf]

Supplementary Information

Wireframe DNA origami for the cellular delivery of platinum(II)-based drugs

Erik De Luca[1], Yang Wang[2], Igor Baars[2], Federica De Castro[1], Marco Lolaico[2], Danilo Migoni[1], Cosimo Ducani[2], Michele Benedetti[1], Björn Högberg[2], Francesco Paolo Fanizzi[1,*]

[1] Dipartimento di Scienze e Tecnologie Biologiche ed Ambientali, Università del Salento, Lecce, Italy.

[2] Division of Medical Systems Bioengineering, Department of Medical Biochemistry and Biophysics, Karolinska Institutet, Solna, Sweden.

[*] Corresponding author.

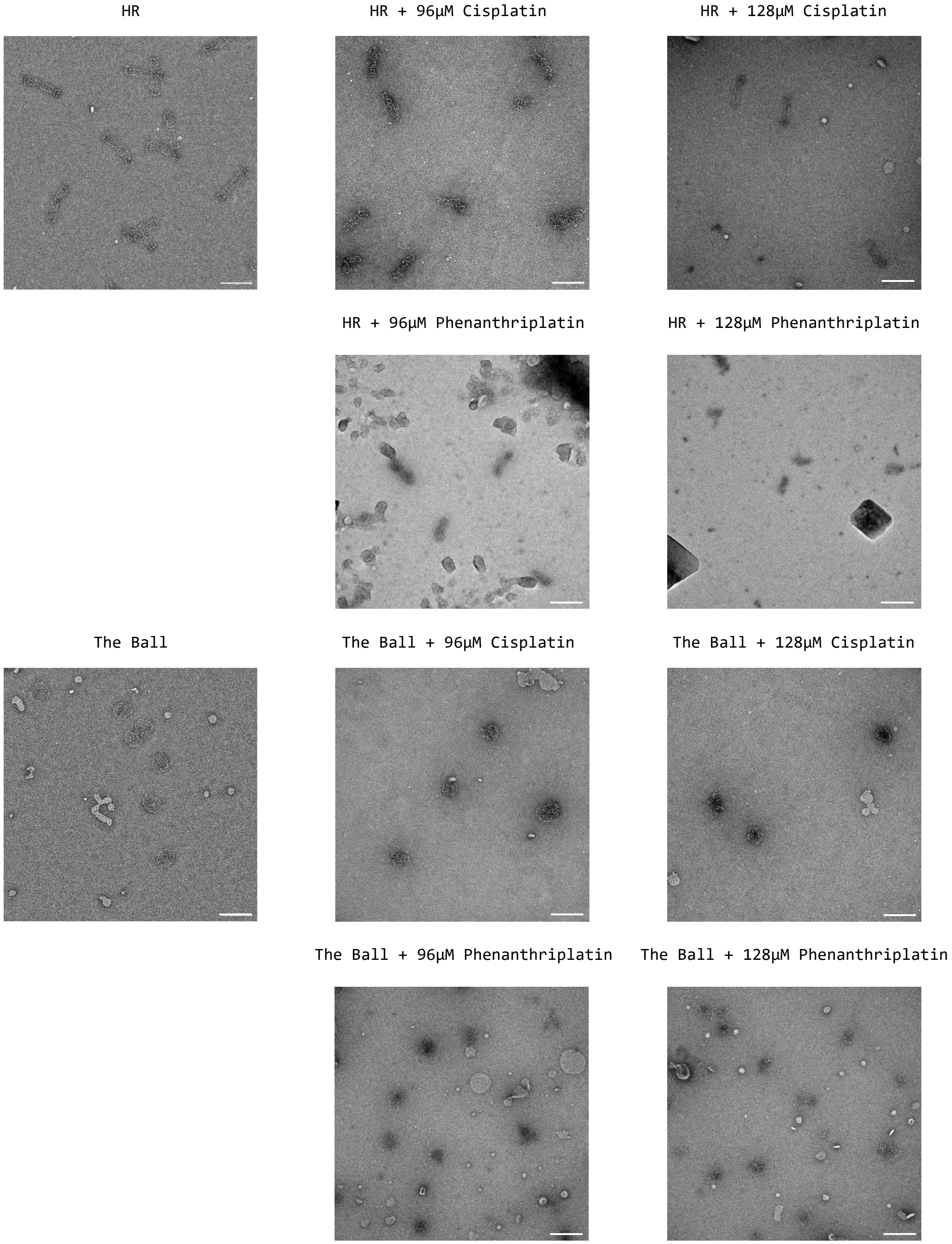

Figure S1. Negative staining TEM of the HR and Ball, comparison with the same structures folded with 96 and 128 μM of cisplatin and phenanthriplatin. Scale bars are 100nm.

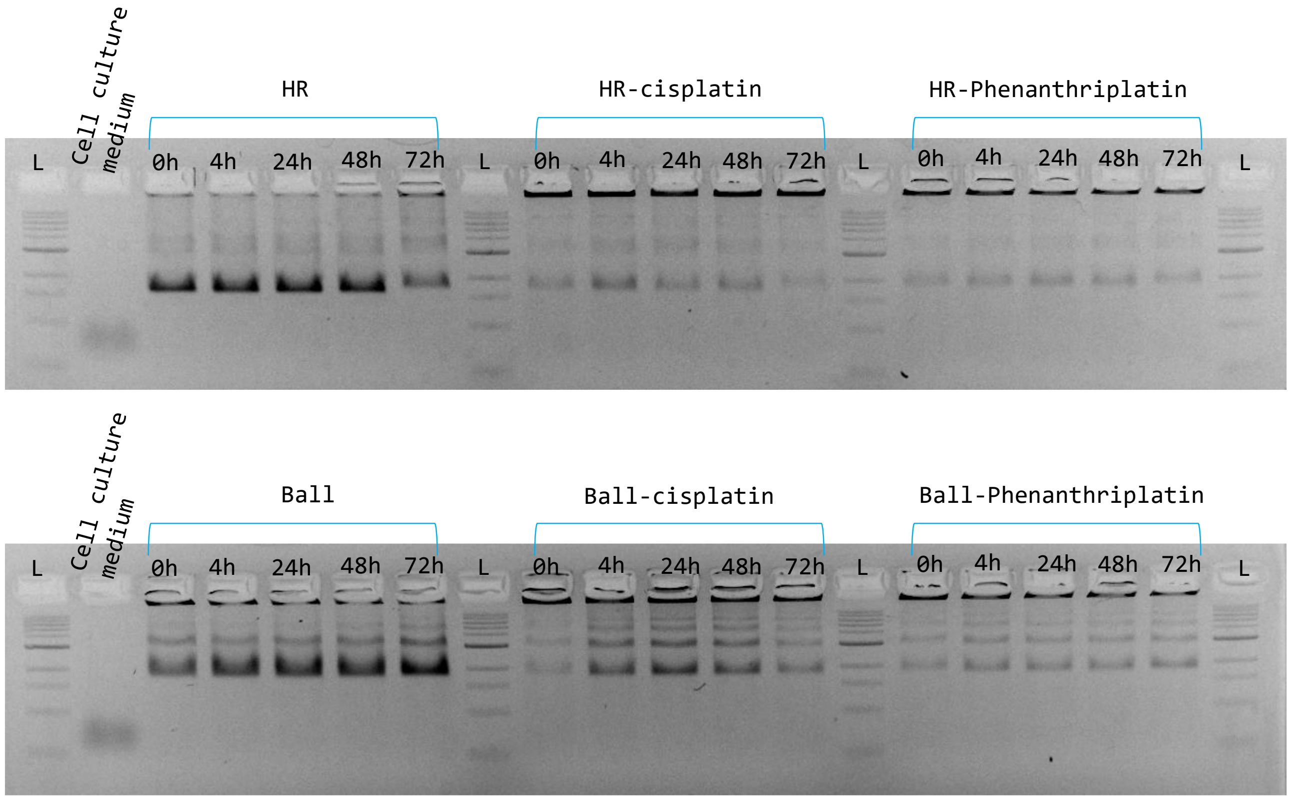

Figure S2. Agarose gel representing the stability of HR and the Ball structures to cell culture media, DMEM containing 10% heat-inactivated fetal bovine serum (FBS). The samples were incubated from 0 to 72 hours at 37℃ and then immediately loaded in a 2% agarose gel supplemented with 10 mM $MgCl_2$ and run for 3 hours at 90 V.

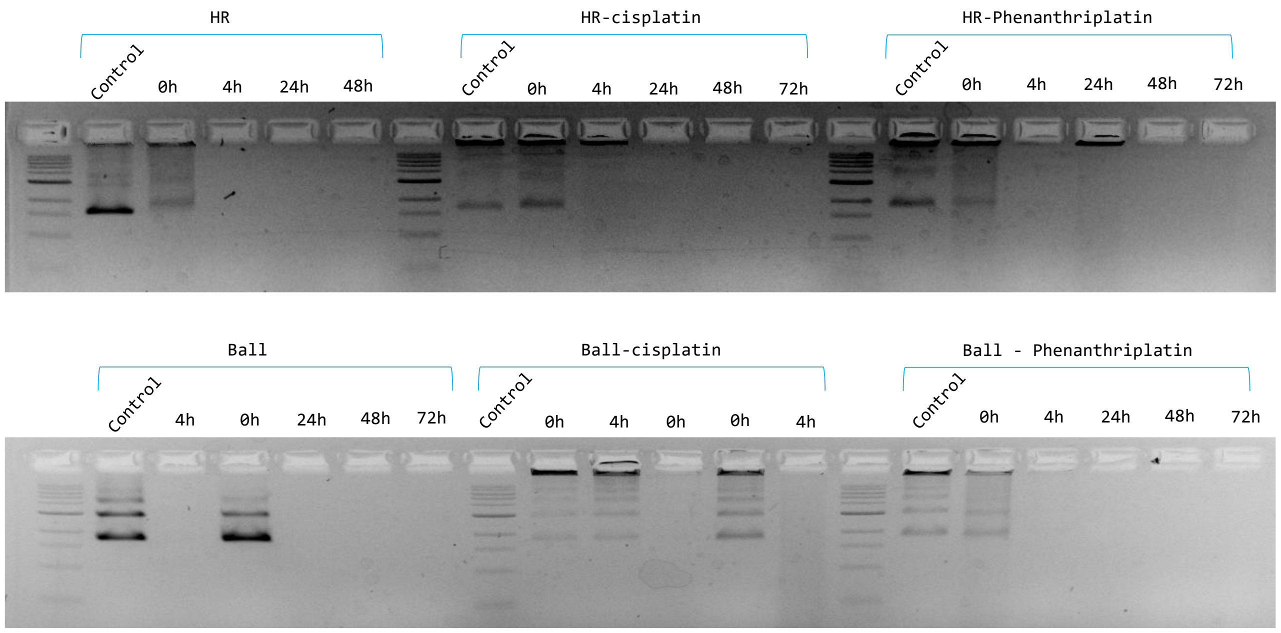

Figure S3. Agarose gel representing the stability of HR and the Ball structures to DNase I. The structures were washed into 1x PBS supplemented with 2.5 mM $MgCl_2$ and 0.1 mM $CaCl_2$. DNase I (New England Biolabs) was diluted in the same buffer and added at a concentration of 0.36 U/ml. The samples were incubated from 0 to 72 hours at 37 ℃ and then immediately loaded in a 2% agarose gel supplemented with 10 mM $MgCl_2$ and run for 3 hours at 90 V.

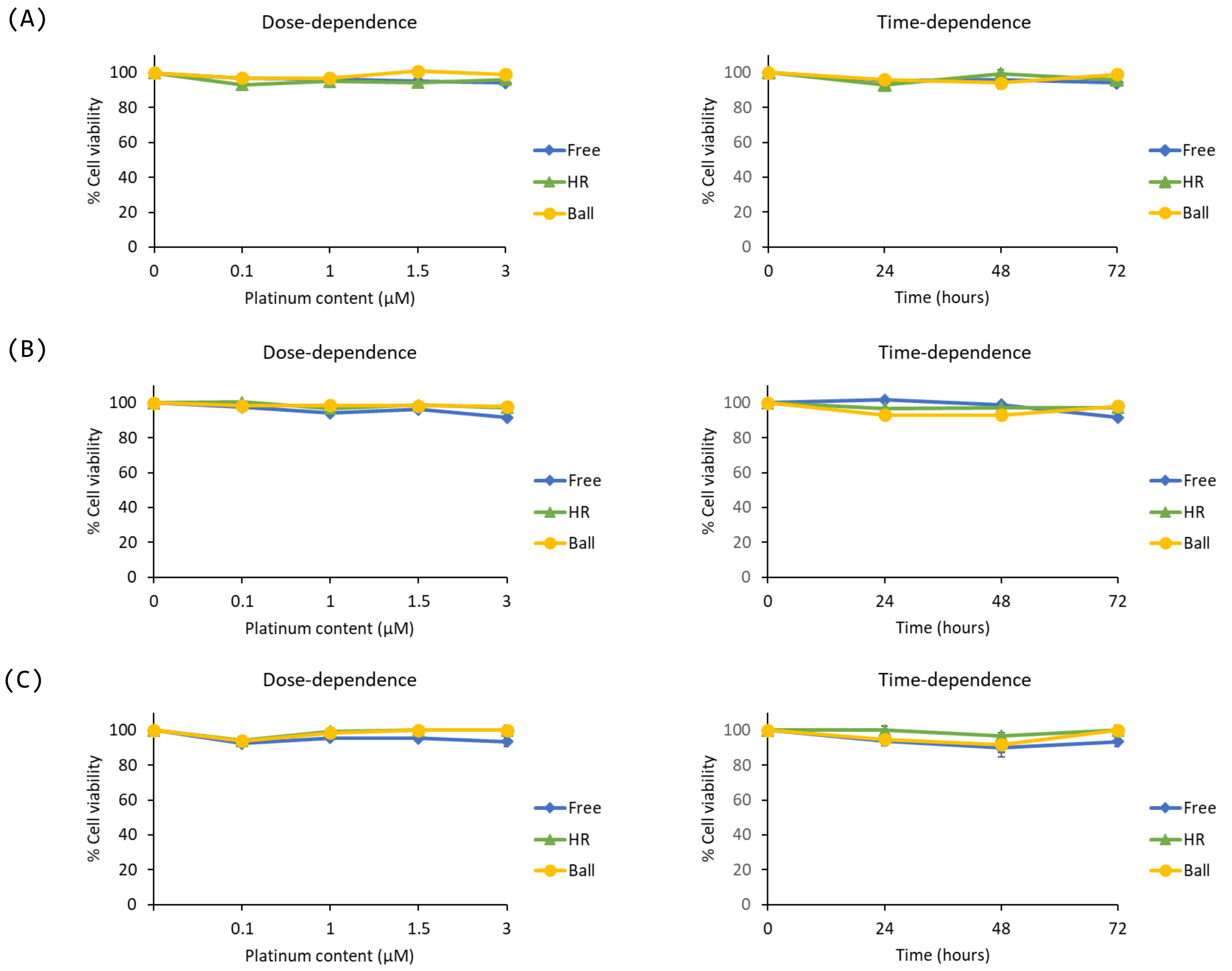

Figure S4. Left panel dose-dependence at 72 hours. Right panel time-dependence at 3 µM of cisplatin loaded nanostructures. A) A549, lung adenocarcinoma cells. B) HeLa, cervical adenocarcinoma cells. C) MCF-7, breast adenocarcinoma cells.
